# The long-term survival of the doublet regimen of concurrent chemoradiation therapy for locoregionally advanced nasopharyngeal carcinoma: a retrospective study

**DOI:** 10.1186/s13014-022-02158-4

**Published:** 2022-11-17

**Authors:** Zhi-Qiang Wang, Xu-Dong Feng, Chun-Lei Ge, Yi Yang, Na Liang, Qing Ye, Yang Fu, Jian Wei, Yong Zhang, Rong-Qing Li

**Affiliations:** 1 Department of Radiation Oncology, First Affiliated Hospital of Kunming Medical University, 295 Xichang Road, Kunming, 650032 People’s Republic of China; 2 Department of Cancer Biotherapy Center, The Third Affiliated Hospital of Kunming Medical University (Tumor Hospital of Yunnan Province), Kunming, 650118 People’s Republic of China

**Keywords:** Intensity-modulated radiation therapy, Locoregionally advanced nasopharyngeal carcinoma, Concurrent chemotherapy

## Abstract

**Objective:**

This study introduces innovative strategies, the doublet regimen of concurrent chemoradiotherapy, to ensure longer survival for locoregionally advanced nasopharyngeal carcinoma.

**Methods:**

We retrospectively reviewed 104 locoregionally advanced nasopharyngeal carcinoma patients who underwent taxane combined platinum-based concurrent chemoradiotherapy in our center between January 2013 and December 2018. All statistical analyses were performed using the Kaplan–Meier method (SPSS 23.0). Different groups were compared with the Wilcoxon rank-sum test.

**Results:**

Ultimately, 104 patients were selected for this study, including 18 and 86 who received either concurrent chemoradiation therapy alone or concurrent chemoradiation therapy plus adjuvant chemotherapy, respectively. The median follow-up time for progression free survival was 53.0 months (IQR 48.5–57.5). The 3-years progression-free survival (PFS), overall survival (OS), local–regional recurrence-free survival (LRRFS) and distant metastasis-free survival (DMFS) rates of the doublet regimen of concurrent chemotherapy for locoregionally advanced nasopharyngeal carcinoma were 85.9%, 96.0%, 96.0% and 90.8%, respectively. Additionally, we analyzed the subgroups and found that the 3-years PFS, OS, LRRFS and DMFS rates for stage III versus stage IVa were 97.8% versus 75.5% (P = 0.000), 100% versus 92.5% (P = 0.004), 100% versus 92.4% (P = 0.015) and 97.8% versus 82.8% (P = 0.002), respectively. During concurrent chemotherapy, acute chemotherapy adverse events of grade 3 or 4 was only 18.3%. Leukopenia was the most common acute chemotherapy adverse event (in 10 patients [9.6%]), followed by neutropenia (in 8 patients [7.6%]).

**Conclusion:**

The doublet regimen of taxane plus platinum concurrent chemoradiotherapy resulted in improved long-term survival of locoregionally advanced nasopharyngeal carcinoma patients, especially for local control rate and warrants further prospective evaluation.

## Introduction

Nasopharyngeal carcinoma (NPC) is a malignant tumor derived from the nasopharyngeal epithelium [1]. Globally, it is estimated that there were 129,079 new cases of NPC among the global cancer cases 2018 [2]. Unlike other cancer types, NPC is prevalent in regions in South Asia, especially for southern China and has unique sensitivity to both radiotherapy and chemotherapy. Single platinum-based concurrent chemoradiation therapy is the backbone of treatment for locoregionally advanced nasopharyngeal carcinoma [3], and the 3-year progression-free survival is merely 70–80% [4–6], which is much lower than 95–100% for early-stage nasopharyngeal carcinoma [7, 8]. This suggests that platinum-based concurrent chemoradiation therapy alone may be insufficient to effectively eliminate micrometastases, and new strategies or methods are required to achieve better survival.

It is crucial to choose a new chemotherapeutic agent that is distinguished from platinum in mechanisms of action and has radiosensitization effects, and combined platinum-based concurrent chemoradiation therapy may further improve the efficacy. Taxane is cycle-specific agent that promotes the assembly of tubulin into microtubules and prevents the dissociation of microtubules, thus preventing mitosis and blocking the cell cycle in the G2/M phase [9], which is the most sensitive phase of radiotherapy. Furthermore, phase I/II clinical trials have also suggested that taxane monotherapy with concurrent chemoradiation therapy for NPC has shown satisfying safety and efficacy [10–12]. The 3-year overall survival, distant metastasis-free survival, and local control rates of simultaneous IMRT and concurrent weekly taxane for locally advanced NPC were 77.0%, 64.4%, and 97.6%, respectively [10]. Taxane combined with platinum-based concurrent chemoradiation therapy is extremely promising to improve the long-term survival of locally advanced NPC.

Based on the above theoretical support and clinical research data, in this study, we retrospectively reviewed and evaluated the overall survival and progression-free survival of locoregionally advanced NPC patients who received taxane combined with platinum-based concurrent chemoradiation therapy in our center.

## Materials and methods

### Patients

We retrospectively reviewed all NPC patients who underwent taxane combined with platinum-based concurrent chemoradiation therapy in our center between January 2014 and December 2018. All patients were restaged according to the 8th edition of the International Union against Cancer/American Joint Committee on Cancer (UICC/AJCC) manual based on their imaging examinations and the previously physical examination.

A total of 330 patients were identified, and eligibility criteria included the following: (1) a pathological diagnosis of NPC; (2) T3N2-3M0 and T4N1-3M0 classification according to the 8th edition of the UICC/AJCC clinical staging system; (3) age ≥ 18 years; (4) Karnofsky Performance Status (KPS) ≥ 80; (5) completion of radical radiotherapy; and (6) received taxane combined with platinum-based concurrent chemoradiation therapy.

The exclusion criteria were as follows: (1) age ≥ 65 years; (2) disease progression during radiotherapy; (3) lack of concurrent chemotherapy or concurrent chemotherapy not platinum-based; (4) received induction chemotherapy or other anti-vascular and immunological agents; and (5) no evidence of previous malignancy or other concomitant malignant disease.

### Chemotherapy

During the study period, concurrent chemoradiation therapy plus adjuvant chemotherapy for stage III–IV disease was recommended according to our institutional guidelines. The study-defined concurrent chemoradiation therapy regimen was docetaxel (75 mg/m^2^ on Day 1) or paclitaxel (135 mg/m^2^ on Day 1) plus nedaplatin (100 mg/m^2^ on Day 2) or cisplatin (80 mg/m^2^ on Day 2) every 3 weeks for 2–3 cycles started on the first day of radiotherapy. The adjuvant chemotherapy regimen was the same as the concurrent chemoradiation therapy regimen every 3 weeks for 2–4 cycles. Dose modifications of concurrent chemotherapy or adjuvant chemotherapy were dependent on the hematological and nonhematological toxicities from the previous cycle. The chemotherapy drug dose was reduced by one level (20% of the original dose) for the first episode of the above toxicities. Patients receiving other chemotherapy regimens or who received only one cycle of concurrent chemotherapy were excluded from this study. All patients were treated with granulocyte colony stimulating factor (G-CSF) on the 2nd day after chemotherapy.

### Radiotherapy

All patients were treated with radical intensity-modulated radiotherapy (IMRT). In general, all patients were immobilized in the supine position using a thermoplastic mask that covered the head, neck, and shoulder. Finally, the patients were scanned by CT from the vertex to the upper margin of the manubrium sternum, with 3-mm slices. Nonenhanced CT and contrast-enhanced CT images were obtained for dose calculation and target delineation, respectively.

According to our institutional guidelines, we defined gross tumor volume (GTV) as the gross tumor determined by physical examination and imaging (including MRI and PET/CT), including GTVnx (gross tumor volumes of the nasopharynx) and GTVnd (gross tumor volumes of the lymph nodes). GTVnx included the primary tumor volume and the enlarged retropharyngeal nodes, while GTVnd was the volume of involved gross cervical lymph nodes. The clinical tumor volume (CTV) includes the primary tumor with potential subclinical disease. The high-risk clinical target volume (CTV1) was defined as the GTVnx plus a 10-mm margin (2–3 mm posteriorly if adjacent to the brain stem or spinal cord) to encompass the high-risk sites of microscopic extension, the whole nasopharynx, retropharyngeal nodal regions and the whole neck area (if there was no positive lymph node in the III–V area, only bilateral levels II, III, and Va were routinely covered). The low-risk clinical target volume (CTV2) was defined as the elective neck area from level IV to Vb and the supraclavicular fossae if there were no positive lymph nodes in the III–V area. The planning target volume (PTV) was created by adding a three-dimensional margin of 3–5 mm to the delineated target volume to compensate for the uncertainties in the treatment setup.

All patients reused a thermoplastic mask that covered the head, neck, and shoulder, The prescribed doses were 70–72 Gy, 68–70 Gy, 60 Gy, and 54 Gy in 33 fractions for the PGTVnx, PGTVnd, PTV1 and PTV2 derived from GTVnx, GTVnd, CTV1, and CTV2, respectively. All patients were treated once daily, with five fractions administered weekly. The doses to organs at risk were within the tolerance range and were calculated to meet the criteria as closely as possible according to the RTOG 0615 protocol.

### Follow-up

The last follow-up date was August 31, 2021. Patient follow-up was measured from the first day of radiotherapy to the last examination or death. Patients were examined every 3 months for one year, every 4–6 months from the second to the third year, and annually thereafter until death. Primary tumors in the nasopharynx and neck were assessed to determine whether local–regional recurrence had occurred by MR. Additional chest CT, abdomen ultrasound/CT or ECT were performed to clarify the patient’s systemic metastasis. The efficacy was jointly evaluated by the radiation oncologist and the imaging doctor. Failures were classified as “in field” if 95% of rGTV was within the 95% isodose, “marginal” if 20% ~ 95% of rGTV was within the 95% isodose, or “outside” if less than 20% of rGTV was inside the 95% isodose [13].

Acute chemotherapy toxicity was evaluated and graded according to the Common Terminology Criteria for Adverse Events version 5.0. As for late radiation toxic effects, we used the late radiation morbidity scoring scheme of the Radiation Therapy Oncology Group (RTOG) to grade it.

### Statistical analysis

The events for progression-free survival (PFS), overall survival (OS), local–regional recurrence-free survival (LRRFS) and distant metastasis-free survival (DMFS) were tumor progression, death from any cause, local and regional relapse, and distant metastasis, respectively. The duration was calculated from the date of radiotherapy to the date of each event or the last follow-up. Survival results were calculated, and all statistical analyses were performed using the Kaplan–Meier method (SPSS 23.0 statistical software, Chicago, IL, USA). Different groups were compared with the Wilcoxon rank-sum test.

## Results

### Clinical characteristics of the patients

**Fig. 1:**
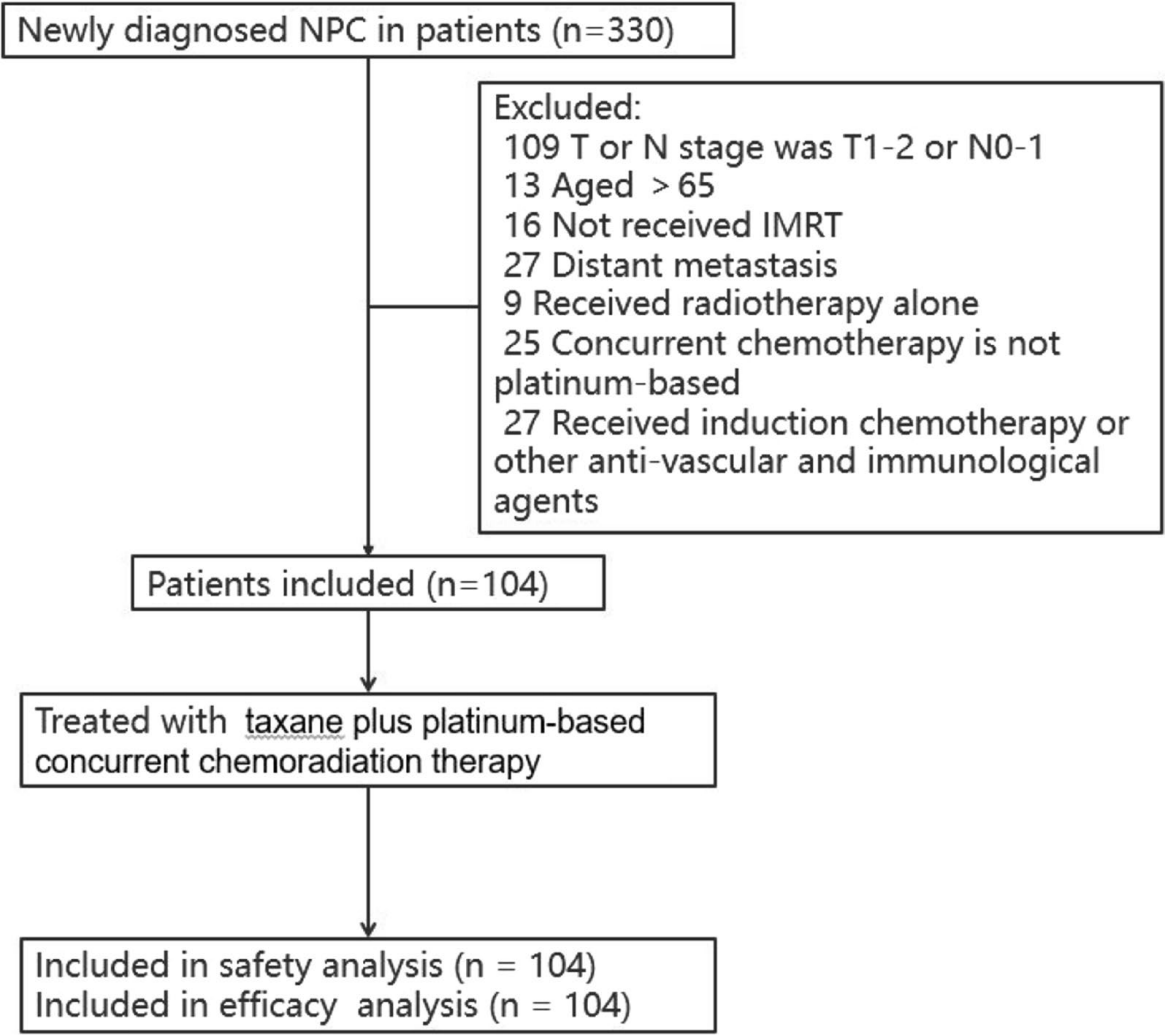
Study flow diagram

Between January 2014 and December 2018, we retrospectively reviewed 330 patients with NPC who underwent taxane combined with platinum-based concurrent chemoradiation therapy in our center. Ultimately, 104 patients were selected for this study (Fig. 1), including 18 and 86 who received either concurrent chemoradiation therapy alone or concurrent chemoradiation therapy plus adjuvant chemotherapy, respectively. The baseline demographics and disease characteristics are shown in Table 1. The median age at the time of radiotherapy was 46.0 (IQR 40.0–54.0) years. Among the 104 patients, 56 (53.8%) were stage IVa patients, and the proportions of T4 and N3 classifications were 80% and 20%, respectively. At a median follow-up of 54.5 months (IQR 44–68 months), the 1-, 3-, and 5-year follow-up rates were 97.1%, 92.3% and 62.5%, respectively.

**Table 1:** Clinical characteristics of the patients

Parameter	All patients (N = 104)
*Sex*	
Female	28
Male	76
*Pathological grade*	
WHO I	2
WHO II	0
WHO III	102
*Age, years*	
Median IQR	46 (40–54)
*T classification*	
T1	0
T2	0
T3	57
T4	47
*N classification*	
N0	0
N1	18
N2	68
N3	18
*Clinical stage*	
III	48
IVa	56
Time of radiotherapy (day)	47.5(45–50)
*Chemotherapy*	
CCRT III IVa	18 10 8
CCRT + Adj III IVa	86 30 56

*NPC* nasopharyngeal carcinoma; *IMRT* intensity-modulated radiotherapy; *RT* radiotherapy; *CCRT* concurrent chemotherapy; *Adj* adjuvant chemotherapy

### Patient survival outcomes

The last follow-up date was August 31, 2021. Eight patients were lost to follow-up, 12 patients died, and 84 patients were alive. The median follow-up time for PFS was 53.0 months (IQR 48.5–57.5). All patients (100%) achieved an objective response, and a complete response was achieved in 86 (88.3%) patients at 3 months post radiotherapy, while 103 (99%) patients achieved a complete response at 6 months post radiotherapy (Table 2).

**Table 2:** Response and survival data

	Taxane combined platinum-based concurrent chemoradiotherapy
Response at 3 months post radiotherapy*	N = 102
Objective Response	102 (100.0%)
Disease control	102 (100.0%)
Complete response	86 (88.3%)
Partial response	17 (16.7%)
Stable disease	0 (0.0%)
Progressive disease	0 (0.0%)
Response at 6 months post radiotherapy	N = 104
Objective response	104 (100.0%)
Disease control	104 (100.0%)
Complete response	103 (99.0%)
Partial response	1 (1.0%)
Stable disease	0 (0.0%)
Progressive disease	0 (0.0%)

*There was 2 patient who did not undergo radiology examination for tumor response evaluation at 3 months post radiotherapy. Percentage values are rounded

**Fig. 2:**
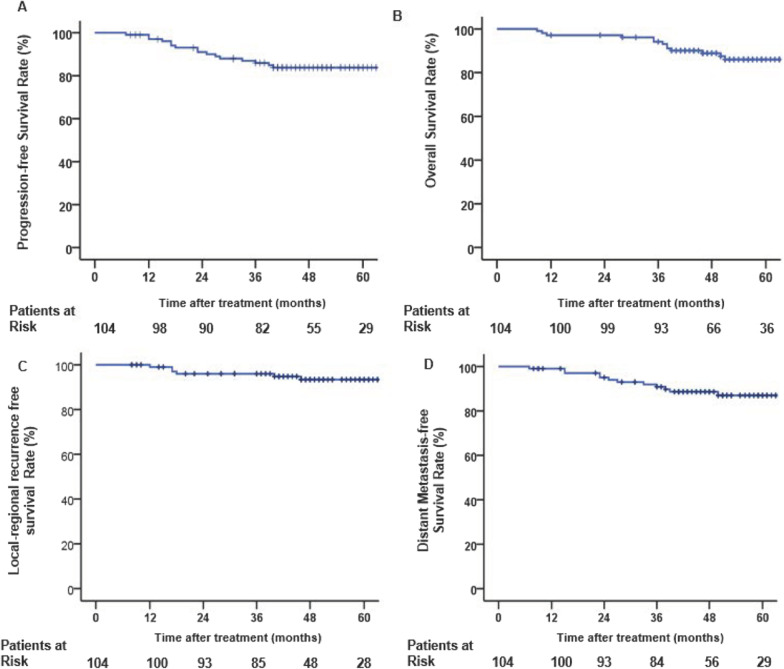
Kaplan–Meier curves of progression-free survival (**A**), overall survival (**B**), locoregional recurrence-free survival (**C**) and distant metastasis-free survival (**D**) for locoregionally advanced NPC with taxane combined with platinum-based concurrent chemoradiation therapy

**Fig. 3:**
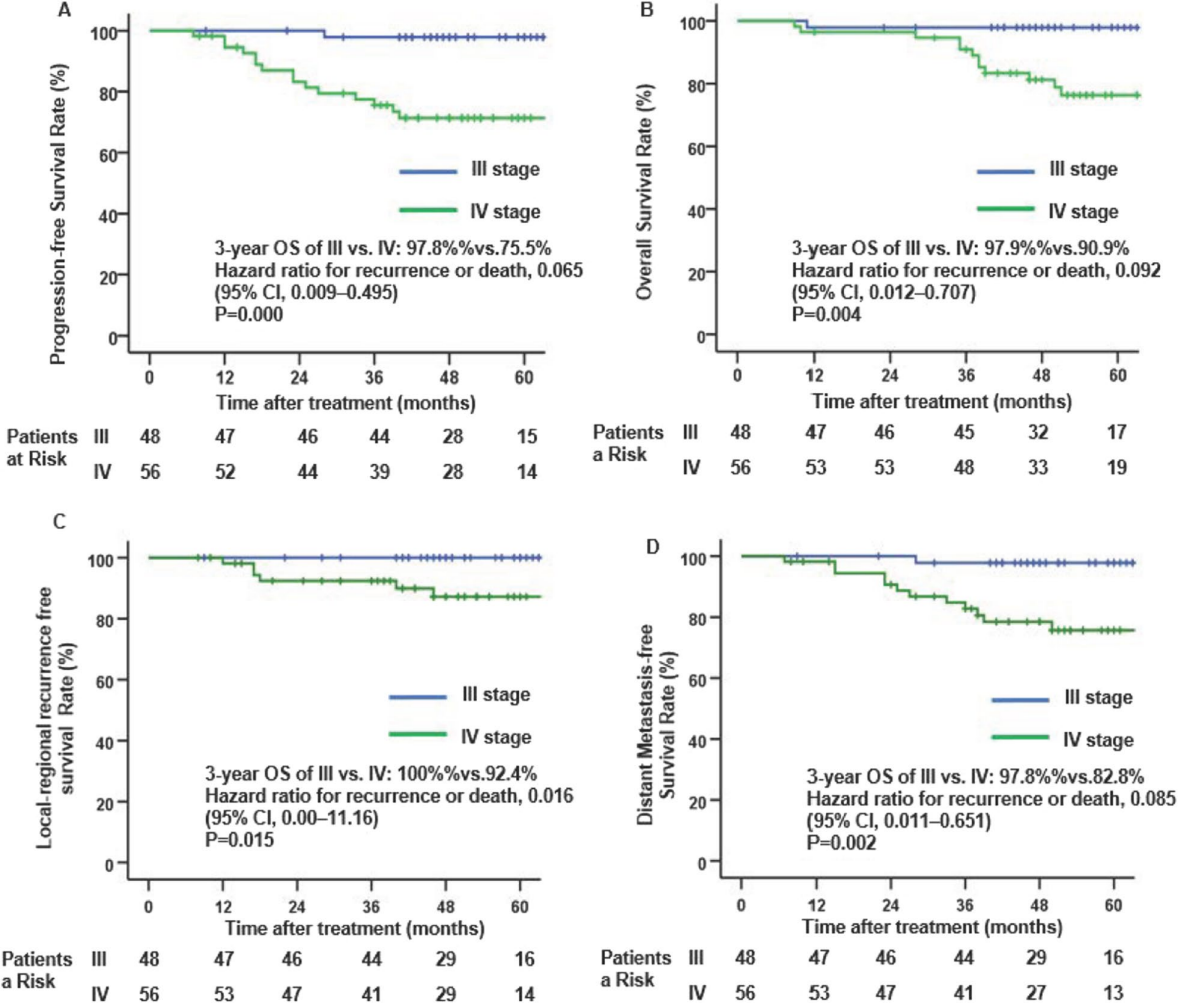
Kaplan–Meier curves of progression-free survival (**A**), overall survival (**B**), locoregional recurrence-free survival (**C**) and distant metastasis-free survival (**D**) for stage III and IV NPC with a doublet regimen of concurrent chemoradiation therapy

Among the 104 patients, 7 patients experienced local regional recurrence, of which one had in-field recurrence and 4 had marginal-field recurrence, followed by salvage IMRT with or without chemotherapy. Twelve patients developed distant metastasis and underwent IMRT for oligometastatic or systemic chemotherapy for multiple metastases, of which only two patients were alive with neoplasm. A total of 13 patients died, of which one patient died of acute myocardial infarction 9 months after the initial radiotherapy, 2 patients developed nasopharyngeal mucosa necrosis and died of hemorrhage from the nasopharynx 11 months after the initial radiotherapy and 8 months after reirradiation, and 10 patients died of distant metastatic lesions (lung and/or liver). The 3-year PFS, OS, LRRFS and DMFS rates of the doublet regimen of concurrent chemotherapy for locoregionally advanced NPC were 85.9%, 96.0%, 96.0% and 90.8%, respectively (Table 3) (Fig. 2), and the 5-year estimated PFS, OS, LRRFS and DMFS rates were 83.8%, 84.7%, 93.4% and 87.0% respectively. Additionally, we analyzed the subgroups and found that the 3-year PFS, OS, LRRFS and DMFS rates for stage III versus stage IVa were 97.8% versus 75.5% (P = 0.001), 100% versus 92.5% (P = 0.004), 100% versus 92.4% (P = 0.015) and 97.8% versus 82.8% (P = 0.002), respectively (Fig. 3).

**Table 3:** The 1,3,5-year survival rates of whole series and different stage

		All (%)	III (T3N2M0) (%)	IVa (T3N3M0 and T4N1-3M0) (%)	Chi-square	P value
PFS	1-year 3-year 5-year	97.1 85.9 83.8	100 97.8 97.8	94.5 75.5 71.3	12.561	0.000
OS	1-year 3-year 5-year	99.0 96.0 84.7	100.0 100.0 100.0	98.1 92.5 72.4	8.305	0.004
LRRFS	1-year 3-year 5-year	99.0 96.0 93.4	100 100 100	98.1 92.4 87.2	5.868	0.015
DMFS	1-year 3-year 5-year	99.0 90.8 87.0	100.0 97.8 97.8	98.2 82.8 75.7	9.160	0.002

All data are presented as No. (%). Percentage values are rounded

### Toxicity

At the time of the last follow-up, all patients were included in the safety analysis. During concurrent chemotherapy, 19/104 patients (18.3%) had acute chemotherapy adverse events of grade 3 or 4 (Table 4). Leukopenia was the most common event (in 10 patients [9.6%]), followed by neutropenia (i n 8 patients [7.6%]). Limited by the study itself, we did not evaluate the acute radiation toxicities. Regarding late adverse events, a total of 85 patients were included in the safety analysis due to the death or loss of some patients (Table 4). The most common adverse events were grade (G) 1–2 xerostomia in 56 (65.9%), followed by G1-2 deafness in 38 (44.7%) patients, G1-2 skin damage in 29 (34.1%) patients, G1-2 temporal-lobe necrosis in 17 (20.0%) patients, G1-2 eye damage in 16 (18.8%) patients, G1-2 dysphagia in 15 (17.6%) patients, G1-2 trismus in 10 (11.8%) patients, and G1-2 nasopharyngeal wall necrosis in 1 (1.2%) patient. We recorded 7 of 85 patients (8.2%) with adverse events of grade 3 to 5. The incidence rates of G3 deafness, trismus and xerostomia were 4.7%, 1.2% and 1.2%, respectively. G4 deafness was observed in 1 (4.0%) patient, who experienced permanent hearing loss. The incidence of G3-4 adverse events was low. However, two patients experienced internal carotid artery rupture and eventually died. Additionally, the mean number of days of radiotherapy was 47.5 (range, 45 to 50), and the normal radiotherapy time was 45 days.

**Table 4:** Acute adverse events and grade for concurrent chemotherapy

Event for acute chemotherapy toxicity	Taxane combined platinum-based concurrent chemoradiotherapy N = 104					
	All grades	Grade 1	Grade 2	Grade 3	Grade 4	Grade 5
Leukopenia	72 (69.2%)	30 (28.8%)	32 (30.8%)	9 (8.7%)	1 (1.0%)	0 (0.0%)
Neutropenia	37 (35.6%)	13 (12.5%)	16 (15.4%)	4 (3.8%)	4 (3.8%)	0 (0.0%)
Anemia	35 (33.7%)	34 (32.7%)	1 (1.0%)	0 (0.0%)	0 (0.0%)	0 (0.0%)
Thrombocytopenia	6 (5.8%)	5 (4.8%)	1 (1.0%)	0 (0.0%)	0 (0.0%)	0 (0.0%)
Hyper creatinine	3 (2.9%)	3 (2.9%)	0 (0.0%)	0 (0.0%)	0 (0.0%)	0 (0.0%)
Total bilirubin elevation	17 (16.3%)	12 (11.5%)	5 (4.8%)	0 (0.0%)	0 (0.0%)	0 (0.0%)
Conjugated bilirubin concentration elevation	16 (15.4%)	10 (9.6%)	6 (5.8%)	0 (0.0%)	0 (0.0%)	0 (0.0%)
Unconjugated bilirubin concentration elevation	16 (15.4%)	11 (10.6%)	5 (4.8%)	0 (0.0%)	0 (0.0%)	0 (0.0%)
Hyponatremia	21 (20.2%)	17 (16.4%)	4 (3.8%)	0 (0.0%)	0 (0.0%)	0 (0.0%)
Hypochloremia	19 (18.3%)	16 (15.4%)	3 (2.9%)	0 (0.0%)	0 (0.0%)	0 (0.0%)
Hypokalemia	19 (18.3%)	15 (14.4%)	3 (2.9%)	1 (1.0%)	0 (0.0%)	0 (0.0%)
Nasopharyngeal wall necrosis	3 (3.5%)	0 (0.0%)	1 (2.4%)	0 (0.0%)	0 (0.0%)	2 (2.4%)
Eye damage	16 (18.8%)	13 (15.3%)	3 (13.5%)	0 (0.0%)	0 (0.0%)	0 (0.0%)
Deafness	43 (50.6%)	17 (20%.0)	21 (24.7%)	4 (4.7%)	1 (1.2%)	0 (0.0%)
Trismus	11 (12.9%)	5 (5.9%)	5 (5.9%)	1 (1.2%)	0 (0.0%)	0 (0.0%)
Xerostomia	57 (67.1%)	30 (35.3%)	26 (30.6%)	1 (1.2%)	0 (0.0%)	0 (0.0%)
Skin damage	29 (34.1%)	23 (27.1%)	6 (7.1%)	0 (0.0%)	0 (0.0%)	0 (0.0%)
Dysphagia	15 (17.6%)	13 (15.3%)	2 (2.4%)	0 (0.0%)	0 (0.0%)	0 (0.0%)
Temporal-lobe necrosis	17 (20.0%)	16 (18.8%)	1 (1.2%)	0 (0.0%)	0 (0.0%)	0 (0.0%)

^§^85 patients were included in the safety analysis due to the death or loss of patients

All data are presented as No. (%). Percentage values are rounded

## Discussion

NPC is sensitive to both radiotherapy and chemotherapy. The 3-year PFS of radiotherapy for early-stage nasopharyngeal carcinoma was 90–100% [7, 8], while the 3-year PFS of single platinum-based concurrent chemoradiation therapy for locoregionally advanced NPC was merely 70–80% [4–6]. Our results revealed that taxane combined with platinum-based concurrent chemoradiation therapy showed promising antitumor activity against locally advanced NPC, with a favorable long-term survival, progression-free survival outcome and tolerated toxicity profile.

This study mainly enrolled locally advanced NPC patients with T3 and T4 disease to minimize the impact of tumor clinical stage on treatment results. Among them, there were 57 patients with stage T3 disease and 47 patients with stage T4 disease. Unfortunately, only 18 people received concurrent chemoradiation therapy, and 86 received concurrent chemoradiation therapy plus 2 to 4 cycles of adjuvant chemotherapy, which may bias the ultimate survival outcome.

Platinum-based concurrent chemoradiation therapy is the backbone of treatment for locoregionally advanced NPC. According to the latest research, the 3-year PFS, OS, locoregional recurrence-free survival (LRFS) and DMFS rates of single platinum-based concurrent chemoradiation therapy alone for locoregionally advanced NPC patients were 76.5%, 90.3%, 91.0% and 84.4%, respectively [3]. However, in our study, the 3-year PFS, OS, LRRFS and DMFS rates were 85.9%, 96.0%, 96.0% and 90.8%, respectively. These results suggest that taxane combined with platinum-based concurrent chemoradiation therapy may be better than platinum-based concurrent chemoradiation therapy alone. Unfortunately, our study is a single-arm retrospective study. A prospective study is needed to further validate the survival benefit of taxane combined with platinum-based concurrent chemoradiation therapy strategies.

Additionally, we conducted a subgroup analysis of stage III and IVa patients and found that the 3-year PFS, OS, LRRFS and DMFS rates for stage III versus stage IVa were 97.8% versus 75.5% (P = 0.001), 100% versus 92.5% (P = 0.004), 100% versus 92.4% (P = 0.015) and 97.8% versus 82.8% (P = 0.002), respectively. The long-term survival of stage III NPC patients was significantly better than that of stage IVa patients. More interestingly, we found that the 3- and 5-year LRRFS and OS rates for stage III NPC were both 100%, and only two patients had distant metastases, which achieved the same therapeutic effect as the treatment of stage I nasopharyngeal carcinoma (5-year LRFS: 100%) [7, 14]. Although the 3- and 5-year LRRFS rates for stage IVa (T3N3M0 and T4N1-3M0) NPC were 92.4% and 87.2%, respectively, which were similar to previous historical data (the 3- and 5-year LRFS rates of induction chemotherapy combined with single platinum-based concurrent chemoradiation therapy and adjuvant chemotherapy for T1-4N3M0 NPC were 92.2% and 89.4%) [15], 31% of the patients were stage T1-2 NPC patients in a historical study [15]. These results suggested the synergistic sensitization effect of taxane combined with platinum-based chemotherapy and made a great contribution to LRRFS.

Other scholars have also performed exploratory studies on the doublet regimen of chemotherapy combined with radiotherapy. The survival rates in our study are longer than those reported in historical data [5, 16, 17], and the reasons are as follows: (1) the prescribed doses were 60 Gy/33 fractions for CTV1, and almost all patients were irradiated in the whole neck lymph node area; and (2) 86/104 (82.7%) patients received 2–4 cycles of adjuvant chemotherapy in this study, which contributed to the long-term survival of locally advanced NPC [18].

For late adverse events, the most common adverse event was xerostomia (65.9%), followed by deafness (44.7%), skin damage (34.1%), and temporal-lobe necrosis (20.0%). The highest incidence of grade 3 late radiotherapy toxicity was deafness at 4.7%. We suspect that this is mainly related to the wide range of tumor lesions in patients, and the auditory system is exposed to radiation doses that exceed the safe dose prescribed by the RTOG. The incidence of skin damage was 34.1%, which is related to the high neck radiation dose (60 Gy) of CTV1, while in other studies, the dosage was 54–56 Gy [5, 19, 20]. In addition, the mean number of days of radiotherapy was 47.5 (range, 45 to 50), and the normal radiotherapy time was 45 days. The patients’ radiotherapy time was prolonged for the following reasons: machine malfunctions and radiation toxicity.

Our findings should also be considered in the context of the limitations of this retrospective study. We found that taxane combined with platinum-based concurrent chemoradiation therapy with or without adjuvant chemotherapy markedly improved the locoregional recurrence-free survival for locally advanced NPC, which even reached a 100% control rate, especially for stage III NPC patients. However, the sample size of the subgroup was relatively small, and the 5-yearS follow-up rate was merely 62.5%. In addition, although the sample of stage III and stage IVa patients with concurrent chemotherapy regimens was balanced, the treatment regimens of all patients were not completely unified, which is a disadvantage in this study. The ideal treatment method should be the same protocol and the same chemotherapeutic drugs and treatment equipment. This will be further improved in our future prospective study to provide higher-level clinical data.

## Conclusion

In conclusion, our study observed that the administration of taxane combined with platinum-based concurrent chemoradiation therapy in stage III–IVa NPC patients showed very promising clinical outcomes, especially for locoregional recurrence-free survival rate. However, additional studies, especially prospective, well-designed, and large-sample clinical studies, are needed.

## Data Availability

The datasets used during the current study are available from the Corresponding author on reasonable request.
